# Description of Process for Stamping Plates by Hydraulic Press

**Published:** 1889-07

**Authors:** E. A. Bogue

**Affiliations:** New York


### DESCRIPTION OF PROCESS FOR STAMPING PLATES BY HYDRAULIC PRESS.[1]

BY E. A. BOGUE, M.D., NEW YORK.

WITHIN a few years a material called Spence metal has been devised. This substance is really sulphur and iron. It melts at about the boiling-point of water, and in process of cooling a stage is reached, just before solidification takes place, at which the mass becomes exceedingly fluid. At this stage it can be poured into an impression of plaster or even Stent's composition.

This circumstance has caused Spence metal to be used in the hydraulic press for the purpose of stamping dental plates, as a steady pressure of almost any power may be had by this means. It is also possible to make the swages and stamp a plate within two hours from the time of taking the impression. A description of a case in hand will perhaps best serve my purpose. In the present instance an impression was taken with Stent's material, and all the

---

[1] Read at the semi-annual meeting of the Massachusetts Dental Society, Boston, June 6, 1889.

rest of the work was done by my friend and assistant Mr. Fred Collett. The impression was chilled with cold water, and sculptor's clay was built up around the margins to the height of one-half inch. A paper could have been wrapped around equally well. The impression then was coated with a solution of soap and water. Into this impression, thus prepared, Spence metal, just before the point of solidification, was poured. This Spence metal was chilled immediately on touching the Stent's composition, so that all contraction took place from the top of the centre downward.

The small die thus made was then provided with three legs made of pins heated and pressed into the metal. These pins held it at just the required height, so that the die, being placed in the middle of the iron ring in which the pressure was to be given, stood at the height required for an additional quantity of Spence metal to be poured into the concavity and around this little die up to the required level. This die, being quite cold, is covered with whiting, and a counter-die of Wood's fusible alloy[1] is poured over it. This fusible alloy melts at a still lower temperature than Spence metal, and it is poured over the male swage by using the heavy iron ring in which the counter-die must remain during the swaging process. This first set of swages being completed, duplicates are made, if required, by taking the impression of the male die and repeating the process of casting the swages as often as may be required.

The Spence metal is exceedingly brittle, so nothing but steady pressure must be permitted. If it should be found necessary to strike blows upon the swage, others must be made of some other material. In the present instance a Babbitt's-metal swage with a tin counter-die was made upon which to break up the plate. The flat plate may be placed between the dies with a bit of glove-kid or rubber dam between the plate and the counter-die, and the flask containing it placed directly in the press. The screw at the top of the press being turned down to give such pressure as is possible from above, the second screw connected with the plunger at the side is then gradually turned inward by means of the large driving-wheel. The manometer is watched, as indicating the amount of pressure that is being given; four hundred pounds to the square centimetre is generally enough, though I have as an experiment run it up to twelve hundred.

---

[1] Composed of fifteen parts of bismuth, eight of lead, four of tin, and three of cadmium. This forms a silvery-white, granular alloy, which becomes soft at 135° F. = 57° C., and fuses at about 145° F. = 63° C.

During the swaging process the plate should be frequently annealed. When finally down, close to the duplicate swages, it receives its last trimming, its last annealing, and is then put upon the original die that was made directly from the impression. When taken from the press after this final pressure, the fit is more perfect than any struck swages can make it.

For suction-plates it is generally necessary to scrape the centre of the plaster impression and not to put in an air-chamber, the fit of the hydraulic-press plates seeming to be as good as the impressions from which they are made.

